# Relationship between dialytic parameters and reviewer confirmed arrhythmias in hemodialysis patients in the monitoring in dialysis study

**DOI:** 10.1186/s12882-019-1212-6

**Published:** 2019-03-05

**Authors:** James A. Tumlin, Prabir Roy-Chaudhury, Bruce A. Koplan, Alexandru I. Costea, Vijay Kher, Don Williamson, Saurabh Pokhariyal, David M. Charytan, on behalf of the MiD investigators and Committees, Don Williamson, Prabir Roy-Chaudhury, James Tumlin, Vijay Kher, Vikranth Reddy, Kowdle Chandrasekhar Prakash, David Charytan, Suresh Chandra Tiwari, Saurabh Pokhariyal, Amber Podoll, Sanjeev Jasuja G. Leslie Walters, Kraig Wangsnes, Alexandru Costea, Selcuk Tombul, Balbir Singh, Brajesh Mishra, Sachin Yalagudri, Abhijeet Shelke, Calambur Narasimhan, A. M. Karthigesan, Abraham Oomman, K. P. Pramod Kumar, Bruce Koplan, Upendra Kaul, Tapan Ghose, Ripen Gupta, Arvind Sethi, Nikhil Kumar, Ramesh Hariharan, Rajnish Sardana, Arif Wahab, N. N. Khanna, Mark Smith, Suresh Kamath, Claude Galphin, Puneet Sodhi, Rajsekara Chakravarthy, Subba Rao Budithi, Finnian McCausland, Sanjeev Gulati, Munawer Dijoo, Upendra Singh, Salil Jain, Vishal Saxena, Gaurav Sagar, David Charytan, Rachel Fissell, Robert Foley, Charles A. Herzog, Peter McCullough, John D. Rogers, James A. Tumlin, Peter Zimetbaum, Manish Assar, Mark Kremers, Wolfgang C. Winkelmayer

**Affiliations:** 1 NephroNet Clinical Research Consortium, Atlanta, GA USA; 2 University of Arizona Health Sciences and Southern Arizona VA Health Care System, Tuscon, AZ USA; 3 Brigham & Women’s Hospital, Boston, MA USA; 4 University of Cincinnati School of Medicine, Cincinnati, OH USA; 5 Medanta Kidney & Urology Institute, Medanta – The Medicity, Gurgaon, India; 6 Southeastern Clinical Research Institute, Augusta, GA USA; 7 Department of Nephrology & Renal Transplantation, HCMCT Manipal Hospital Pvt. Ltd, Dwarka, Delhi India; 8 Nephrology Division, New York University School of Medicine and NYU Langone Medical Center, New York, NY USA

**Keywords:** End stage renal disease, Hemodialysis, Cardiovascular disease, Arrhythmia

## Abstract

**Background:**

Hemodialysis patients have high rates of sudden death, but relationships between serum electrolytes, the dialysis prescription, and intra-dialytic shifts in fluid and electrolyte with arrhythmia are uncertain.

**Methods:**

We analyzed sixty-six hemodialysis patients who underwent loop recorder implantation with continuous electrocardiographic monitoring, weekly to bi-weekly testing of pre- and post-dialysis electrolytes, and detailed capture of dialysis prescription and flow sheet data for 6 months. The incidence rate ratio (IRR) of reviewer confirmed arrhythmias (RCA) during dialysis through 8 h after dialysis and associations with serum chemistries and dialytic parameters were assessed using adjusted, negative-binomial regression.

**Results:**

Among 66 individuals with a mean age of 56 years, 12,480 events were detected in 64 (97%) patients. RCA nadired 12–24 h after dialysis and increased during the final 12 h of the inter-dialytic interval through the first 8 h after dialysis. Higher pre-dialysis serum magnesium concentration was associated with lower incidence rate ratio for arrythmia (IRR per 1 mg/dL increase 0.49, 95% CI; 0.25, 0.94), as was dialysate calcium concentration > 2.5 mEq/L vs. 2.5 mEq/L (IRR 0.52, 95% CI: 0.39, 0.70). Neither intradialytic serum potassium nor weight change were significantly associated with RCA rate. However, there was effect modification such that arrhythmia rate was maximal with concurrently high intradialytic volume and potassium removal (P_interaction_ = 0.01).

**Conclusions:**

Intra and post-dialytic arrhythmias are common in hemodialysis. Additional studies designed to further elucidate whether modification of the serum magnesium concentration, dialysate calcium concentration, and the extent of intradialytic potassium and fluid removal reduces the risk of per-dialytic arrhythmia are warranted.

**Trial registration:**

Clinicaltrials.gov NCT01779856. Prospectively registered on January 22, 2013.

**Electronic supplementary material:**

The online version of this article (10.1186/s12882-019-1212-6) contains supplementary material, which is available to authorized users.

## Background

Patients on dialysis experience a disproportionately high rate of cardiovascular morbidity and mortality with 3-year mortality of nearly 50%. Multiple studies demonstrate that cardiovascular disease is the most common cause of death, and approximately two thirds of the cardiac deaths are consistently attributed to arrhythmias [1–4]. This increased incidence of cardiovascular disease, particularly of sudden death, is not fully explained by traditional cardiovascular risk factors [5, 6] and appears to be unique to end stage renal disease.

Interestingly, previous studies have shown a clear relationship between the dialysis cycle and sudden death, with both cardiac deaths and sudden deaths most likely to occur following the long, 3-day inter-dialytic interval [7, 8]. Observational data, have also demonstrated associations between serum electrotype concentrations or the dialysate electrolyte concentration and the risk of sudden death [9–12].

These observations suggest current approaches to thrice-weekly dialysis could potentially induce cardiac arrhythmias. However, previous studies analyzing associations between dialytic parameters and the occurrence of arrhythmia have primarily been limited to retrospective analyses of dialysis organization data with large numbers of clinical events but without detailed electrocardiographic tracings, frequent laboratories, or detailed capture of the dialysis prescription. Conversely, the few studies with electrocardiographic data, were constrained by the limits of available technology to short-term capture of arrhythmia data over a maximal observation period of 1–3 dialysis sessions.

To better understand associations between hemodialysis and the occurrence of arrhythmia we analyzed data from the the Monitoring in Dialysis (MiD) study [13], which used implantable loop recorders (ILR) to continuously capture cardiac rhythm over 6 months in patients receiving maintenance hemodialysis.

## Methods

### Study population

The design, objectives, and primary outcomes of the Monitoring in Dialysis (MiD) study have been reported previously [13, 14]. MiD (NCT01779856) was a prospective, multi-center study conducted in the US and India between January 2013 and September 2015 designed to characterize arrhythmias occurring in three times-weekly hemodialysis dialysis patients. An implantable cardiac monitoring device (Medtronic Reveal® XT or LINQ) was inserted to record heart rate and rhythm. Major inclusion criteria included the following: a) age ≥ 21 years and b) receiving in-center hemodialysis 3 times/week or with estimated glomerular filtration < 15 mL/min/1.73m^2^ and expected to start dialysis within 2 months (no pre-dialysis patients were enrolled). Individuals with permanent pacemakers or implantable defibrillators and those not expected to remain on in-center dialysis for at least 6 months due to poor prognosis, expected transplant, or change in modality were excluded. All patients provided written informed consent, and institutional review board or ethics committee approval was obtained at each participating center. The trial was terminated when the last enrolled patient had completed the 6-month primary observation period.

### Study procedures

ILR were downloaded and vital signs, dialysis prescription, and dialysis flow-sheet parameters were recorded at each dialysis session for 6 months. In addition, downloads were performed after any dialysis session with a blood draw for a total of up to 5 downloads each week. Programming parameters used for automated detection included the following: a) Fast ventricular tachycardia—180 beats per minute (BP) for 30/40 beats; b) ventricular tachycardia—130 BPM for 5 beats; c) bradycardia—40 BPM for 4 beats; d) asystole—duration of 3 s; e) atrial fibrillation detection on with no minimal duration.

Serum chemistries were tested before and after dialysis twice weekly for 4 weeks and then weekly through 6 months. ILR tracings that were marked by patients as symptomatic and those with potential arrhythmias were reviewed by the study sponsor by at least one trained individual with experience in cardiac signal interpretation and adjudication. Those potentially meeting the primary study endpoint, were subsequently adjudicated by a core lab.

### Definitions

The primary study endpoint of clinically significant arrythmia (CSA) has been previously reported [14] and included ventricular tachycardia > 115 beats/minute (modified by protocol amendment to improve detection specificity to ≥130 beats per minute) lasting ≥30 s, bradycardia with rate ≤ 40 beats per minute lasting ≥6 s, asystole for ≥3 s, and patient-marked (symptomatic) events where ECG review showed an arrhythmia considered clinically relevant. We report here, the secondary endpoint of reviewer confirmed arrhythmia (RCA). RCA was defined as an ILR identified or patient marked event in which a manual review of the stored ECG tracing confirmed the presence of abnormal rhythm RCA included outcomes meeting the primary endpoint definition, atrial fibrillation, supraventricular tachycardia, and sinus tachycardia with rate exceeding 130 beats per minute. In addition, ECG-confirmed ventricular tachycardia, asystole, and bradycardia of insufficient rate or duration to meet the primary endpoint definition were included. As for the primary endpoint of CSA, the RCA endpoint was chosen to define a set of events representing clinically relevant electrical instability and chronotropic dysfunction likely to share common physiologies and thus meriting joint analysis. ECG reviewers were not aware of patients characteristics or dialysis prescriptions. .

### Statistical analyses

Baseline demographic, dialysis parameters, and laboratory characteristics are presented as mean ± standard deviation (SD), and median interquartile range (IQR) for continuous variables and percent (n/N) for categorical variables. Distributions in baseline characteristics were analyzed according to quartiles of the observed number of RCA events during follow-up in the primary analysis and according the dichotomous presence or absence of RCA during follow-up in a secondary analysis. Time averaged serum electrolyte concentrations or dialysis prescription parameters are presented as the mean or median of all sessions depending on normality. Characteristics between subjects with and without at least one RCA were compared using an unpaired t-test or Wilcoxon rank sum test for continuous variables, and Fisher’s exact tests for categorical variables. Where 3 or more groups were assessed ANOVA and Kruskal Wallis tests were used for continuous variables and Fisher’s exact test was used for categorical variables.

Negative binomial mixed-effect regression was used to analyze RCA rate over the dialytic week with division into 3 intra-dialytic periods and 14 inter-dialytic blocks of 12 h each. Repeated measures within subjects were accounted for using a random intercept that included an offset to indicate the time within each period. We excluded any off-schedule dialysis sessions as well as week in which dialysis was performed more or less than 3 times because the primary aim was to assess associations with the thrice weekly dialysis schedule. We used the lowest frequency period as the reference period for pairwise comparisons.

Given that dialytic parameters are most likely to directly induce arrythmia during dialysis or within a few hours after the conclusion of dialysis (and less likely to be responsible the further out one gets from dialysis), we investigated differences between sessions with RCA during the interval including dialysis or the 8 h immediately after dialysis using mixed effect models for continuous measures, or logistic regression (binary or multinomial) for categorical measures. The random effect of subject was included in both models. The interval represented the peak period of intra/post-dialysis arrythmia and was therefore the time period during which RCA were most likely to be biologically influenced by the dialysis procedure.

Negative binomial mixed effect regression was used to analyze associations of electrolytes, dialysis prescription, and intradialytic changes in fluid or electrolyte (flux, the difference between pre and post dialysis values) with RCA during the interval beginning with the start of each dialysis session through 8 h after dialysis. A random intercept was included to account for within-subjects repeated measures. Multivariable analyses were adjusted for age, sex, race, dialysis vintage, and vascular access and incidence rate ratios (IRR) were calculated. To avoid over-specification of the models, electrolyte and dialytic parameters of interest were added individually to this base model. First order interactions between volume removal (expressed as intra-dialytic weight change) and dialysate electrolyte concentration were analyzed to assess for effect modification by the extent of ultrafiltration given an extensive literature connecting ultrafiltration and risk of death [15]. All analyses were completed using SAS v 9.4 (Cary, NC) with *P* < 0.05 considered significant.

## Results

### Baseline characteristics and RCA

Overall, RCA were observed in 64 (97%) subjects during following-up (Table 1). Among individuals with ≤10, 11–49, 50–239 and ≥ 240 RCA events during follow-up, mean age was 61.1 ± 9.1, 56.3 ± 12.0, 54.1 ± 13.0 and 54.1 ± 13.7 years (*P* = 0.31). Other characteristics at baseline, such as race, sex, systolic and diastolic blood pressures, cause of ESRD, ESRD vintage, history of diabetes, and history of heart failure also did not differ significantly across quartiles of observed number of RCA events (*P* ≥ 0.14 for all comparisons). Similarly, there were no significant differences in baseline characteristics among those with (*n* = 64) and without (*n* = 2) RCA (Additional file 1: Table S1).

**Table 1:** Baseline characteristics according to the number of reviewer confirmed arrythmias during follow-up

Characteristics	All Subjects (*N* = 66)	Number of RCA During Follow-up				*P* Value
		≤10 (*N* = 16)	11–49 (*N* = 16)	50–239 (*N* = 17)	≥240 (*N* = 17)	
Age (years)	56.3 ± 12.2	61.1 ± 9.1	56.3 ± 12.0	54.1 ± 13.0	54.1 ± 13.7	0.31
Female	20/66 (30.3%)	5/16 (31.3%)	5/16 (31.3%)	5/17 (29.4%)	5/17 (29.4%)	> 0.99
Race						0.15
Asian	23/66 (34.8%)	9/16 (56.3%)	7/16 (43.8%)	4/17 (23.5%)	3/17 (17.6%)	
Black	35/66 (53.0%)	7/16 (43.8%)	8/16 (50.0%)	11/17 (64.7%)	9/17 (52.9%)	
Other	1/66 (1.5%)	0/16 (0.0%)	0/16 (0.0%)	0/17 (0.0%)	1/17 (5.9%)	
White	7/66 (10.6%)	0/16 (0.0%)	1/16 (6.3%)	2/17 (11.8%)	4/17 (23.5%)	
Systolic blood pressure	140.8 ± 23.4	140.9 ± 19.9	145.9 ± 23.9	135.8 ± 23.0	140.9 ± 27.0	0.68
Diastolic blood pressure	80 (70, 84)	76 (59, 80)	80 (71, 81)	80 (64, 85)	84 (74, 90)	0.14
Weight (kg)	81.7 (68.2, 95.2)	78.1 (68.8, 91.0)	84.8 (61.7, 89.5)	80.4 (68.9, 119.0)	83.7 (75.8, 108.2)	0.59
BMI ≥ 40	6/66 (9.1%)	1/16 (6.3%)	1/16 (6.3%)	2/17 (11.8%)	2/17 (11.8%)	> 0.99
Cause of ESRD						
Diabetes	28/66 (42.4%)	11/16 (68.8%)	6/16 (37.5%)	4/17 (23.5%)	7/17 (41.2%)	0.14
Glomerulonephritis	6/66 (9.1%)	0/16 (0.0%)	1/16 (6.3%)	4/17 (23.5%)	1/17 (5.9%)	
Hypertension	25/66 (37.9%)	5/16 (31.3%)	6/16 (37.5%)	8/17 (47.1%)	6/17 (35.3%)	
Other	10.6% (7/66)	0/16 (0.0%)	3/16 (18.8%)	1/17 (5.9%)	3/17 (17.6%)	
ESRD Vintage (years)	2.4 (1.2, 5.3) (*N* = 65)	2.2 (1.2, 4.3) (*N* = 16)	2.9 (1.5, 5.5) (*N* = 15)	2.5 (1.2, 5.7) (*N* = 17)	2.5 (0.7, 5.3) (*N* = 17)	0.78
Prior kidney transplant	9/66 (13.6%)	2/16 (12.5%)	4/17 (23.5%)	3/17 (17.6%)	2/16 (12.5%)	0.25
Previous peritoneal dialysis	7/66 (10.6%)	1/16 (6.3%)	2/16 (12.5%)	2/17 (11.8%)	2/17 (11.8%)	> 0.99
Vascular Access						
AV Fistula	45/65 (69.2%)	12/16 (75.0%)	12/16 (75.0%)	11/17 (64.7%)	10/16 (62.5%)	0.87
AV Graft	17/65 (26.2%)	3/16 (18.8%)	3/16 (18.8%)	5/17 (29.4%)	6/16 (37.5%)	
Catheter	3/65 (4.6%)	1/16 (6.3%)	1/16 (6.3%)	1/17 (5.9%)	0/16 (0.0%)	
Diabetes	42/66 (63.6%)	13/16 (81.3%)	10/16 (62.5%)	8/17 (47.1%)	11/17 (64.7%)	0.25
Hyperlipidemia	40/66 (60.6%)	9/16 (56.3%)	8/16 (50.0%)	11/17 (64.7%)	12/17 (70.6%)	0.66
Hypertension	56/66 (84.8%)	15/16 (93.8%)	13/16 (81.3%)	15/17 (88.2%)	13/17 (76.5%)	0.61
Ischemic heart disease	32/66 (48.5%)	10/16 (62.5%)	8/16 (50.0%)	8/17 (47.1%)	6/17 (35.3%)	0.51
History of MI	6/66 (9.1%)	1/16 (6.3%)	2/16 (12.5%)	1/17 (5.9%)	2/17 (11.8%)	0.95
Congestive heart failure	17/66 (25.8%)	3/16 (18.8%)	3/16 (18.8%)	4/17 (23.5%)	7/17 (41.2%)	0.46
Coronary artery bypass surgery	9/66 (13.6%)	4/16 (25.0%)	3/16 (18.8%)	1/17 (5.9%)	1/17 (5.9%)	0.32
Arrhythmia	21/66 (31.8%)	3/16 (18.8%)	4/16 (25.0%)	7/17 (41.2%)	7/17 (41.2%)	0.42
Smoking						0.45
Current	5/66 (7.6%)	0/16 (0.0%)	1/16 (6.3%)	3/17 (17.6%)	1/17 (5.9%)	
Never	46/66 (69.7%)	13/16 (81.3%)	9/16 (56.3%)	11/17 (64.7%)	13/17 (76.5%)	
Past	15/66 (22.7%)	3/16 (18.8%)	6/16 (37.5%)	3/17 (17.6%)	3/17 (17.6%)	
LVEF	55.0 (55.0, 60.0) (N = 65)	55.0 (55.0, 60.0) (*N* = 15)	57.8 (55.0, 63.5) (*N* = 16)	60.0 (55.0, 63.0) (*N* = 17)	55.0 (55.0, 60.0) (*N* = 17)	0.65

Basline characteristics in individuals with and without reviewer confirmed arrhythmia (RCA) during follow-up. *BMI* body mass index, *ESRD* end stage renal disease, *AV* arterio-venous, *LVEF* Left ventricular ejection fraction. Data are mean ± SD, median (IQR), or n/N (%)

Among multiple baseline pre-dialysis laboratory tests examined, few differed significantly across quartiles of observed RCA (Table 2). However, use of high flux dialyzer was more frequent among those with a higher number of RCA (31.3, 56.3, 76.5, and 88.2% among those with ≤10, 11–49, 50–239, and ≥ 240 RCA, *P* = 0.004). Dialysate chemistries at baseline were similar across quartiles of observed RCA, but there was a non-significant trend towards increased use of higher dialysate potassium concentrations among those with more RCA (*P* = 0.05). Findings were similar when analyzed according to the dichotomous presence or absence of RCA during follow-up. with exception that the dialysate calcium concentration which was significantly higher (2.5 [IQR: 2.5, 2.5] vs. 1.6 [IQR: 1.6, 1.6], *P* = 0.04) in those with compared to those without RCA (Additional file 1: Table S2).

**Table 2:** Laboratory values and dialysis prescription according to the number of reviewer confirmed arrythmias during follow-up

Characteristics	All Subjects (*N* = 66)	Number of RCA During Follow-up				*P* Value
Laboratory Parameters		≤10 (*N* = 16)	11–49 (*N* = 16)	50–239 (*N* = 17)	≥240 (*N* = 17)	
Blood Urea Nitrogen (mg/dL)^a^	59.7 ± 17.8	63.6 ± 22.5	53.4 ± 17.0	63.6 ± 14.5	59.1 ± 17.6	0.39
Sodium (mEq/L)^a^	138.0 (135.0, 140.0)	137.0 (132.5, 138.5)	137.0 (136.0, 139.5)	139.0 (135.5, 140.0)	140.0 (135.0, 141.0)	0.41
Potassium (mEq/L)^a^	4.7 (4.2, 5.4)	5.3 (4.6, 5.9)	4.5 (4.2, 5.1)	4.6 (4.2, 5.1)	4.7 (4.4, 5.4)	0.32
CO2 (mEq/L)^a^	22.2 ± 3.7	20.5 ± 3.4	22.6 ± 2.4	22.7 ± 3.5	22.5 ± 4.9	0.38
Calcium (mg/dL)^a^	8.7 ± 0.8	8.5 ± 0.8	8.9 ± 0.7	8.8 ± 0.8	8.6 ± 1.1	0.49
Magnesium (mg/dL)^a^	2.3 (2.0, 2.7)	2.6 (2.2, 3.1)	2.5 (2.1, 2.8)	2.3 (2.1, 2.7)	2.0 (1.7, 2.3)	0.003
Phosphorous (mg/dL)^a^	5.1 (4.3, 6.3)	5.4 (4.5, 6.6)	5.1 (4.2, 5.7)	5.5 (4.4, 6.6)	4.9 (4.2, 7.7)	0.84
Hemoglobin (g/dL)^b^	10.7 (9.9, 11.4)	10.5 (9.8, 10.8)	10.6 (9.3, 11.4)	10.5 (9.8, 11.3)	11.2 (11.1, 11.6)	0.06
Albumin (g/dL)^a^	4.0 (3.8, 4.2)	3.8 (3.6, 4.1)	4.1 (3.9, 4.2)	4.0 (3.8, 4.2)	3.9 (3.8, 4.2)	0.32
spKt/V^a^	1.5 (1.2, 1.7)	1.2 (1.1, 1.7)	1.5 (1.2, 1.7)	1.6 (1.4, 1.7)	1.5 (1.4, 1.7)	0.48
Dialysis Parameters						
Duration of hemodialysis (hrs)	4.0 (3.5, 4.0)	4.0 (3.5, 4.0)	3.9 (3.5, 4.0)	4.0 (3.5, 4.0)	4.0 (3.5, 4.0)	0.99
Dry weight target (kg)	80.5 (65.0, 94.0)	74.3 (66.3, 90.3)	81.5 (61.0, 88.5)	75.0 (65.0, 115.0)	82.0 (73.4, 107.5)	0.55
Kg Over dry weight target	4.2 (2.7, 5.2)	4.0 (2.5, 5.4)	3.5 (1.8, 4.9)	4.7 (2.8, 5.1)	4.3 (3.1, 5.5)	0.44
Ultrafiltration rate (ml/kg/hr)	10.9 (7.4, 15.9)	11.4 (7.7, 13.2)	10.7 (6.3, 16.0)	10.9 (7.5, 15.9)	11.0 (9.2, 15.9)	0.84
Sodium modeling	9/66 (13.6%)	4/16 (25.0%)	2/16 (12.5%)	1/17 (5.9%)	2/17 (11.8%)	0.47
High flux dialyzer	42/66 (63.6%)	5/16 (31.3%)	9/16 (56.3%)	13/17 (76.5%)	15/17 (88.2%)	0.004
Membrane reuse	18/66 (27.3%)	4/16 (25.0%)	3/16 (18.8%)	4/17 (23.5%)	7/17 (41.2%)	0.55
Cellulose membrane	5/66 (7.6%)	3/16 (18.8%)	2/16 (12.5%)	0/17 (0.0%)	0/17 (0.0%)	0.06
Dialysate temperature (°C)	37.0 (37.0, 37.0)	37.0 (36.8, 37.0)	37.0 (37.0, 37.0)	37.0 (37.0, 37.0)	37.0 (37.0, 37.0)	0.52
Dialysate sodium (mEq/L)^c^	140.0 (140.0, 140.0)	140.0 (138.0, 140.0)	140.0 (140.0, 140.0)	140.0 (140.0, 140.0)	140.0 (140.0, 140.0)	0.30
Dialysate potassium (mEq/L)^d^	2.0 (2.0, 2.0)	2.0 (2.0, 2.0)	2.0 (2.0, 2.0)	2.0 (2.0, 2.0)	2.0 (2.0, 3.0)	0.05
Dialysate bicarbonate (mEq/L)	35.0 (33.0, 36.0)	35.0 (32.5, 35.0)	35.0 (32.0, 36.0)	35.0 (35.0, 37.0)	35.0 (35.0, 40.0)	0.33
Dialysate calcium (mEq/L)	2.5 (2.5, 2.5)	2.5 (1.6, 2.5)	2.5 (2.1, 3.0)	2.5 (2.5, 2.5)	2.5 (2.5, 2.5)	0.44

Distribution of baseline lab tests and dialysis parameters. Continuous variables are presented as mean ± standard deviation or median (interquartile range). CO2-total carbon dioxide (bicarbonate)

*SpKt/V* single pool Kt/V according to Daugirdas formula, *CRP* HS-high sensitivity CRP, *PTH* parathyroid hormone, *Hrs* hours, *mEq* milliequivalent, *mg* milligram, *dL* deciliter, *L* liter, *pg* picogram, Celsius, *mL* milliliter, *Kg* kilogram, CRP, PTH and BNP were measured in US patients only

^a^ Available in 59 patients (≤10 RCA (n = 12), 11–49 RCA (n = 16), 50–240 RCA (n = 16) > 240 RCA (n = 15). ^b^Available in 56 patients (≤10 RCA (*n* = 12), 11–49 RCA (n = 14), 50–240 RCA (n = 17) > 240 RCA (*n* = 13). ^c^ Dialysate sodium was available in 61 patients overall (≤10 RCA (*n* = 14), 11–49 RCA (n = 14), 50–240 RCA (*n* = 17) > 240 RCA (*n* = 16). ^d^ Mean values for potassium in mEq/L = 1.9 ± 0.3, 2.3 ± 0.6, 2.2 ± 0.4, and 2.4 ± 0.5

### RCA and dialytic parameters over time

Given the high proportion of patients with RCA during follow-up, we analyzed the difference between sessions with and without RCA. There were 4154 sessions without RCA and 605 sessions with RCA during dialysis or during the 8 h after dialysis. As shown in Table 3, dialysis sessions without RCA were characterized by lower ultrafiltration rates (9.9 ± 4.9 vs. 10.1 ± 4.6 ml/kg/hour, *P* = 0.01) and intra-dialytic decrease in weight (2.6 ± 1.3 vs. 3.0 ± 1.3 kg *P* = 0.002) as well being closer to the target dry weight. There were no significant differences in pre-dialysis electrolyte concentrations or intra-dialytic change in electrolytes in sessions with or without RCA. However, dialysate temperature was lower in sessions without RCA (*P* < 0.001) and use of dialysate with 2.5 mEq/L of calcium was more common in sessions with RCA. Similar associations were present for dialysate temperature but not for indices of fluid accumulation or ultrafiltration rate when analyzing sessions with reviewer confirmed bradyarrhythmias (Additional file 1: Table S3). Differences in dialysate calcium were qualitatively similar but did not achieve significance. In contrast, both indices of fluid gain and removal as well dialysate calcium and temperature were different in sessions without and with reviewer confirmed tachyarrhythmias (Additional file 1: Table S4).

**Table 3:** Characteristics of sessions with and without RCA during follow-up

Characteristic	Sessions without RCA	Session with RCA			*P* Value
	Number of Sessions	Mean ± SD or Median (IQR)	Number of Sessions	Mean ± SD or Median (IQR)	
Duration of hemodialysis (hrs)	4154	3.8 ± 0.5	605	3.9 ± 0.6	0.80
Dry weight (kg)	4150	84.8 ± 27.9	605	90.1 ± 30.2	0.83
Percent over dry weight (%)	4138	3.9 ± 2.4	604	3.9 ± 2.4	0.04
Kilogram over dry weight	4138	3.1 ± 1.9	604	3.6 ± 2.1	0.07
Ultrafiltration rate (ml/kg/hr)	4154	9.9 ± 4.9	605	10.1 ± 4.6	0.01
Intradialytic decrease in weight (kg)	4138	2.6 ± 1.3	604	3.0 ± 1.3	0.002
Pre-dialysis potassium (mEq/L)	1408	4.9 ± 0.8	259	5.0 ± 0.8	0.74
Intradialytic potassium change (mEq/L)	1364	−1.2 ± 0.8	253	− 1.3 ± 0.8	0.13
Pre-dialysis calcium (mEq/L)	1416	8.7 ± 0.9	260	8.8 ± 0.8	0.56
Intra-dialytic calcium change (mEq/L)	1366	0.7 ± 1.1	254	0.6 ± 0.9	0.53
Pre-dialysis magnesium (mg/dL)	1416	2.4 ± 0.5	260	2.2 ± 0.4	0.88
Intradialytic magnesium change (mg/dL)	1370	−0.3 ± 0.3	255	0.3 ± 0.3	0.97
Pre-dialysis phosphorus (mg/dL)	1414	5.3 ± 1.7	260	5.1 ± 1.7	0.31
Intradialytic phosphorus change (mg/dL)	1362	−3.0 ± 1.5	255	−3.0 ± 1.5	0.28
Pre-dialysis bicarbonate (mEq/L)	1415	22.1 ± 3.9	260	22.7 ± 4.2	0.87
Intradialytic bicarbonate change (mEq/L)	1368	4.9 ± 3.3	255	4.5 ± 3.5	0.75
Pre-dialysis sodium (mEq/L)	1416	136.6 ± 4.3	260	137.9 ± 4.1	0.16
Intradialytic sodium change (mEq/L)	1366	0.6 ± 4.1	254	0.0 ± 3.4	0.28
Nadir intradialytic systolic blood pressure (mm Hg)	4148	120.0 (105.0, 138.0)	605	113.0 (99.0, 130.0)	0.31
Nadir intradialytic diastolic blood pressure (mm Hg)	4148	67.0 (56.0, 74.0)	605	62.0 (54.0, 71.0)	0.06
Dialysis Prescription Parameters	Number of Sessions (*n/N*)	%	Number of Sessions (*n/N*)	%	
Dialysate temperature					< 0.001
36–36.9 °C	541/4064	13.3%	46/596	7.7%	
≥37 °C	3523/4064	86.7%	550/596	92.3%	
Dialysate potassium					0.26
2.0 mEq/L	3327/4008	83.0%	422/595	70.9%	
3.0 mEq/L	681/4008	17.0%	173/595	29.1%	
Dialysate calcium					< 0.001
< 2.0 mEq/L	925/4146	22.3%	31/604	5.1%	
2.0–2.4 mEq/L	81/4146	2.0%	1/604	5.1%	
2.5 mEq/L	2211/4146	53.3%	433/604	71.7%	
> 2.5 mEq/L	929/4146	22.4%	139/604	23.0%	
Dialysate sodium					0.67
≤ 135 mEq/L	501/3803	13.2%	70/558	12.5%	
136–139 mEq/L	408/3803	10.7%	21/558	3.8%	
140 mEq/L	2894/3803	76.1%	467/558	83.7%	
Sodium modeling	581/4140	14.0%	48/603	8.0%	0.40
Dialysate bicarbonate					0.20
≤ 28 mEq/L	215/4043	5.3%	29/600	4.8%	
29–34 mEq/L	962/4043	23.8%	125/600	20.8%	
35 mEq/L	1548/4043	38.3%	217/600	36.2%	
> 35 mEq/L	(1318/4043)	32.6%	229/600	38.2%	

Data are presented as number of sessions over mean ± standard deviation or as % (n/N)>. *Hrs* hours, *Kg* kilogram, *mL* milliliter, *mg/dL* milligram per deciliter, *mm Hg* millimeters of mercury

Time averaged laboratory and dialysis prescription parameters were analyzed to assess their distribution according to the number of RCA during follow up. (Additional file 1: Tables S5 & S6). Serum chemistries were notable for lower serum magnesium concentration over time in those with more RCA with concentrations of 2.6, 2.3, 2.3 and 2.2 mg/dL among those with ≤10, 11–49, 50–239, and ≥ 240 RCA, *P* = 0.01). Among dialysis parameters analyzed none were significantly different across categories of the numbers of observed RCA during follow-up. There were no other significant differences between serum chemistries or dialysis prescription parameters among individuals with and without RCA during follow-up.

### RCA rate

**Fig. 1:**
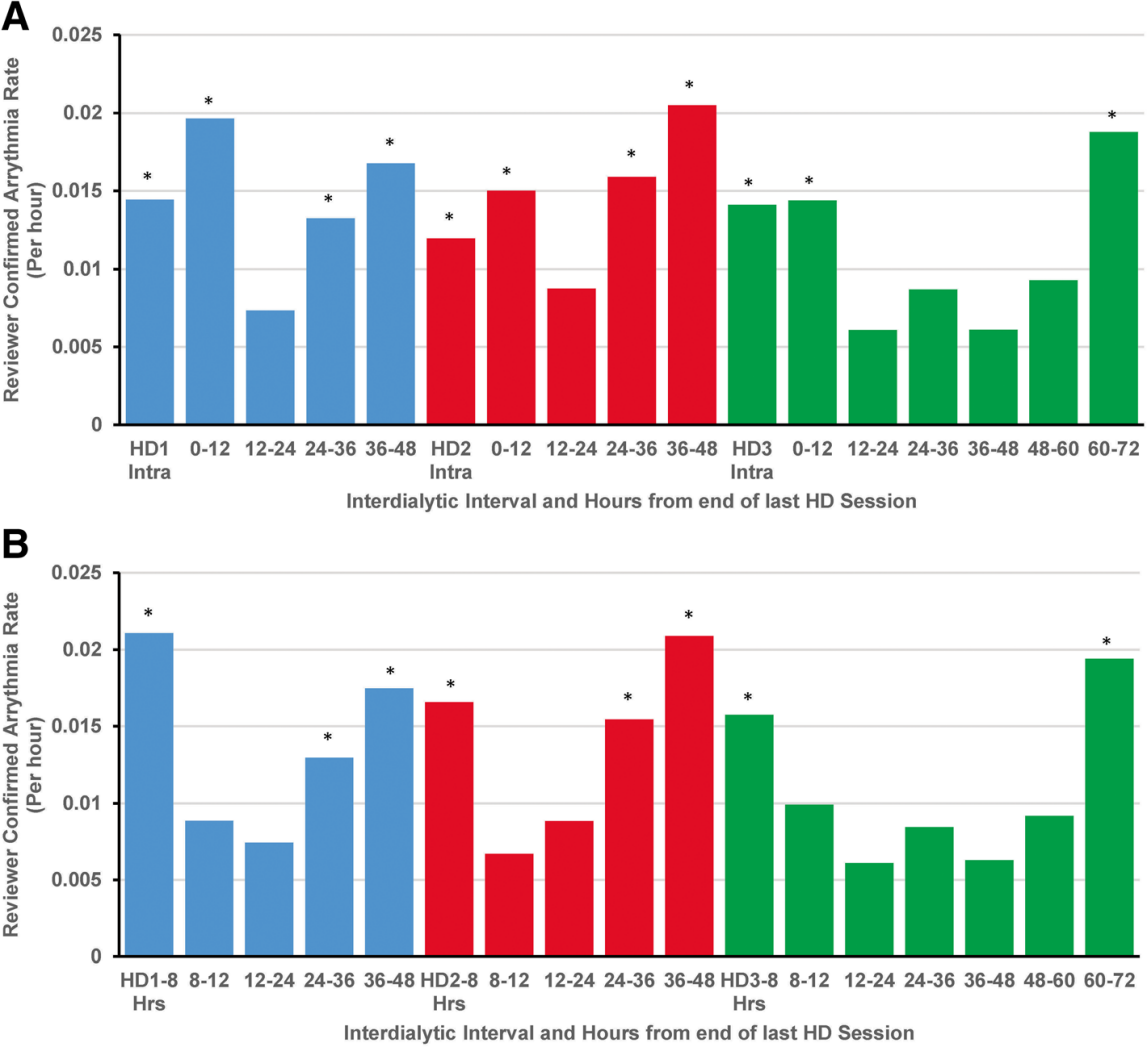
Rate of reviewer confirmed arrhythmia during and between dialysis sessions-- Reviewer confirmed arrhythmia rate over the course of the dialytic week. **a** RCA during the intradialytic interval or successive 12-h intervals from 1 session until the next dialysis session. HD1–3 Intra-1st, 2nd or 3rd, intradialytic interval of the week. **b** RCA during the intradialytic interval through 8 h after dialysis, 8–12 h after dialysis, or successive 12-h intervals until the next dialysis session. HD1–8, HD2–8, HD3–8—1st, 2nd or 3rd, intradialytic interval of the week through 8 h post dialysis. Blue, red, green-1st 2nd or 3rd dialysis session and subsequent inter-dialytic interval of the week. * signifies *P* < 0.05 compared to nadir rate for the week

As reported previously there 12,480 RCA detected in 64 (97%) patients at an overall rate of 33.7 (95% CI: 23.4, 48.7) per patient/month. Multiple episodes demonstrated more than a single type of arrythmia on the tracing, and the total included 7488 atrial arrythmias, 913 ventricular arrythmias, 1770 bradycardia events, 31 asystole, and 6065 sinus tachycardias. The atrial arrythmias included 4419 atrial fibrillation events [16]. There were clear temporal patterns with a nadir occurring 12–24 h after dialysis and an increase in rate during the last 12 h of the inter-dialytic interval through 12 h after dialysis (Fig. 1a). Additional, post-hoc exploration demonstrated that the majority of post-dialysis RCA occur within 8 h after dialysis, and that RCA rate during the next 4 h is not significantly different from that during the nadir period at 12–24 h after 3rd weekly session **(**Fig. 1b).

**Fig. 2:**
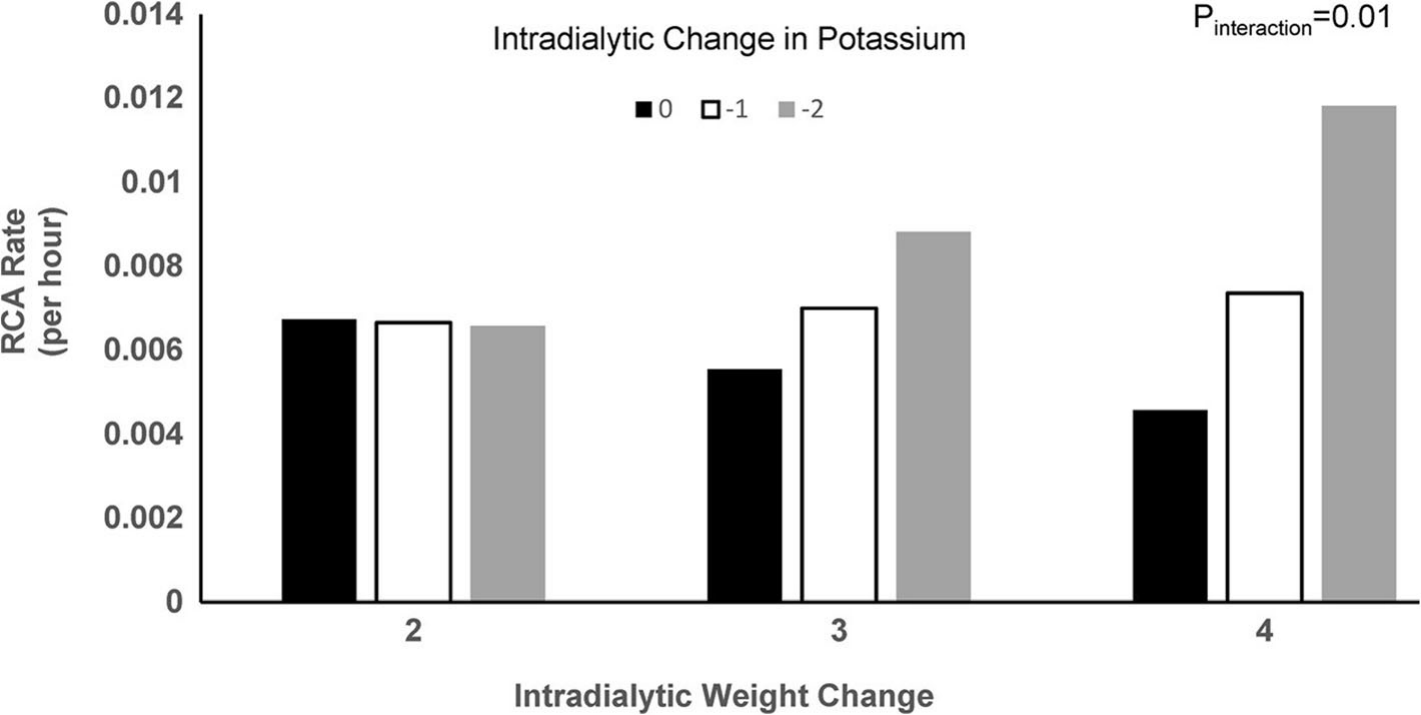
Incidence rate of reviewer confirmed arrhythmias according to intradialytic change in potassium and weight—Rate of reviewer confirmed arrhythmias per hour according to intra-dialytic change in potassium and the pre-post dialysis decrease in weight from the start of dialysis to 8 h post. The interaction term for intradialytic change in weight*intradialytic change in potassium was estimated as an IRR of 0.78 (95% CI: 0.66, 0.93)

Given this distribution and noting that biologically dialytic parameters are most likely to directly induce arrythmia during dialysis or within a few hours after the conclusion of dialysis (and less likely to be responsible the further out one gets from dialysis), we hypothesized the effects of pre-dialysis electrolytes, the dialysis prescription, and the dialysis procedure would be most apparent during dialysis or the 8 h immediately (the period with maximal arrythmia incidence rate). Crude and adjusted associations with RCA rate during dialysis and the 8 h immediately afterwards are shown in Table 4**.** Only 4 parameters were associated with RCA rate in adjusted analyses: Higher pre-dialysis magnesium concentrations were associated with lower risks of RCA (IRR 0.49, 95% CI: 0.25, 0.94). Conversely, dialysate calcium concentrations below 2.0 mEq/L (IRR 0.13, 95% CI: 0.03, 0.57) and above 2.5 mEq/L were associated with lower risks of RCA compared to 2.5 mEq/L dialysate (IRR 0.52, 95% CI: 0.39, 0.70). Dialysate sodium concentrations of 135–139 mEq/L were also associated with a reduced rate compared to dialysate sodium of 140 mEq/L (IRR 0.40, 95% CI: 0.17, 0.95) as were dialysate bicarbonate concentrations > 35 mEq/L compared to concentrations of 35 mEq/L (IRR 0.51, 95% CI: 0.27, 0.97). Other factors were not significantly associated with RCA rate. Lastly, there was evidence for significant interaction between intradialytic change in potassium and change in weight (P_interaction_ = 0.01)—whereas RCA rate changed minimally with potassium flux when ≤2 kg were removed, there were sharp increases in RCA rate with greater potassium flux with ≥3 kg of ultrafiltration (Fig. 2).

**Table 4:** Associations of electrolytes and dialysis parameters with RCA rate from the beginning of each dialysis session to 8-h post-dialysis

Parameter	Crude IRR (95% CI)	*P* Value	Adjusted IRR (95% CI)	*P* Value
Electrolyte Concentration				
Pre-dialysis potassium	1.26 (0.96, 1.65)	0.10	1.28 (0.97, 1.69)	0.08
Intradialytic potassium change	0.78 (0.60, 1.01)	0.06	0.78 (0.60, 1.01)	0.06
Pre-dialysis calcium	0.92 (0.65, 1.31)	0.65	0.80 (0.56, 1.15)	0.23
Intra-dialytic calcium change	1.06 (0.83, 1.34)	0.65	1.16 (0.90, 1.49)	0.26
Pre-dialysis magnesium	0.41 (0.21, 0.78)	0.01	0.49 (0.25, 0.94)	0.03
Intradialytic magnesium change	1.29 (0.57, 2.94)	0.54	1.56 (0.64, 3.78)	0.33
Pre-dialysis phosphorus	1.06 (0.89, 1.26)	0.53	1.00 (0.84, 1.19)	0.96
Intradialytic phosphorus change	0.95 (0.80, 1.14)	0.58	1.02 (0.84, 1.23)	0.86
Pre-dialysis bicarbonate	1.01 (0.95, 1.07)	0.78	0.97 (0.93, 1.03)	0.28
Intradialytic bicarbonate change	0.98 (0.92, 1.05)	0.62	0.99 (0.93, 1.06)	0.80
Sodium pre-dialysis	1.07 (0.99, 1.15)	0.08	1.04 (0.97, 1.11)	0.25
Intradialytic sodium change	0.97 (0.92, 1.03)	0.34	0.98 (0.93, 1.04)	0.54
Dialysis Prescription Parameters				
Dialysate temperature ≥ 37 vs. 36–36.9 °C	3.87 (0.95, 15.78)	0.06	4.30 (0.97, 19.17)	0.06
Dialysis potassium 3 vs. 2 mEq/L	1.23 (0.63, 2.61)	0.49	1.12 (0.59, 2.13)	0.73
Dialysate calcium				
< 2 vs. 2.5 mEq/L	0.11 (0.04, 0.31)	< 0.001	0.13 (0.03, 0.57)	0.01
2.0–2.4 vs. 2.5 mEq/L	0.11 (0.00, 7.30)	0.30	0.09 (0.00, 10.50)	0.32
> 2.5 vs. 2.5 mEq/L	0.54 (0.36, 0.83)	0.004	0.52 (0.39, 0.70)	< 0.001
Dialysate Sodium				
≤ 135 vs. 140 mEq/L	2.31 (0.59, 9.01)	0.23	2.31 (0.60, 8.93)	0.23
135–139 vs. 140 mEq/L	0.38 (0.12, 1.21)	0.10	0.40 (0.17, 0.95)	0.04
Sodium modelling (vs. fixed sodium)	0.57 (0.21, 1.58)	0.28	0.77 (0.37, 1.59)	0.47
Dialysate bicarbonate				
≤ 28 vs. 35 mEq/L	0.25 (0.04, 1.69)	0.15	0.39 (0.07, 2.34)	0.31
> 28–34 vs. 35 mEq/L	0.43 (0.18, 1.06)	0.07	0.96 (0.30, 3.07)	0.95
> 35 vs. 35 mEq/L	0.74 (0.36, 1.52)	0.41	0.51 (0.27, 0.97)	0.04
Intradialytic weight change (per Kilogram)	1.16 (1.02, 1.31)	0.02	1.10 (0.99, 1.22)	0.08
Nadir SBP	1.00 (0.99, 1.01)	0.93	0.99 (0.98, 1.00)	0.21
Nadir DBP	1.01 (0.99, 1.02)	0.35	1.00 (0.98, 1.02)	0.98

Crude and adjusted associations with RCA rate from the start of one dialysis to 8 h after post dialysis. Adjusted models include age, sex, race, vascular access type, and dialysis vintage. All sessions are included. Kg-kilogram. mL-milliliter. mEq/L-milliequivalent/liter. Pre- and intradialytic potassium, calcium, bicarbonate and sodium per 1 mEq/L increase in electrolyte. Pre and intradialytic magnesium and phosphorous per 1 mg/dL change

Results were qualitatively similar for reviewer confirmed bradyarrhythmias and tachyarrhythmias when they were analyzed separately with 3 exceptions: Higher pre-dialysis phosphorous was associated with lower rates of tachyarrhythmia (IRR 0.45, 95% CI: 0.23, 0.91), higher pre-dialysis bicarbonate was associated with lower rates of bradycardia (IRR 0.73, 95% CI: 0.55, 0.98), and bradycardia was less frequent with large intradialytic weight change (IRR 0.58, 95% CI: 0.40, 0.83).

## Discussion

We studied 66 hemodialysis patients with implantable loop recorders to characterize arrhythmias and their relationship to the dialysis procedure during a prolonged monitoring period. We found that 97% of patients experienced an EKG-verified arrhythmia during follow-up. RCA were detected at a rate exceeding 1 event per patient-day of follow-up, and they were most frequent during and shortly after dialysis as well during the last hours of the inter-dialytic window. Although arrhythmias were frequent and clearly tracked the dialytic cycle, among modifiable factors several related to the dialysis prescription including pre-dialysis magnesium, the dialysate calcium concentration, sodium, and bicarbonate concentrations and the combination of high levels of ultrafiltration and potassium flux during dialysis were significantly associated with RCA rate.

Sudden cardiac death is the single most important cause of mortality in hemodialysis patients and is responsible for almost a third of mortality. While it’s clear that the temporal profile of sudden death mirrors the dialysis cycle with a preponderance of events related to the long inter-dialytic period [7], our overall knowledge base remains incomplete with regards to the specific role of the dialysis procedure (with its significant fluid and electrolyte fluxes) in the pathogenesis of this condition. The current analysis of the Monitoring in Dialysis (MiD) study provides important ancillary information demonstrating increased risks for arrhythmia during the peri-dialytic period and identifies several modifiable factors that may represent targets for interventional studies seeking to lower the risk of arrhythmia and sudden death in hemodialysis patients.

Several retrospective cohort studies have demonstrated that the risk of cardiovascular death, cardiovascular hospitalizations and sudden death increase following the long interdialytic interval [7, 8]. These data suggest that increases in the incidence of sudden death in dialysis patients are not solely the result of uremia-induced changes in the myocardium, but instead reflect the ability of the aphysiologic nature of intermittent hemodialysis to trigger the spread of arrhythmias in this vulnerable substrate. More recently, several studies have implicated the dialysis prescription—specifically the use of high ultrafiltration rates or dialysates with low concentrations of potassium or calcium [9, 10, 15, 17].

Nevertheless the underlying arrhythmias responsible for sudden death or indeed whether the majority of sudden deaths are attributable to fatal arrhythmia versus myocardial infarction or non-cardiac causes remains uncertain [18]. Although these studies provide compelling evidence linking the dialysis prescription to sudden death, a major drawback to each is the absence of electrocardiographic data demonstrating the presence of an underlying arrhythmia in cases of sudden death or an increase in arrhythmias in response to changes in these dialysis parameters. Several more recent studies have used implantable loop monitoring technology to confirm that arrhythmias are common in hemodialysis patients and occur in a pattern mimicking the observed patterns of sudden death in registry-based analyses [14, 19, 20], and in the study by Buitten, atrial fibrillation was more common following sessions with lower dialysate potassium concentrations or higher ultrafiltration volumes.

Our analysis extends upon these finding in several ways. We recorded detailed information about the dialysis prescription, blood pressure, and ultrafiltration at every session for 6 months, and we tested blood chemistries using a central lab both before and after dialysis at least weekly for 6 months and bi-weekly during the first month after ILR insertion. To our knowledge, the previous investigations using long-term monitoring during dialysis only recorded the clinical laboratory data (generally drawn once per month), recorded dialysis parameters on only a subset of dialysis sessions, and had no information on post-dialysis electrolyte concentrations. Thus, our findings are based on much richer and more detailed data on the dialysis prescription, peri-dialytic parameters (such as fluid removed), and serum electrolytes. Furthermore, to our knowledge, the current study is unique in having with the data needed to assess the impact of electrolyte flux on arrhythmia.

Although we did not identify a significant association between dialysate potassium or fluid removed and the incidence of arrhythmia, we did find a significant interaction demonstrating that arrhythmia rate increased as the amount of volume removed and the change in potassium during dialysis increased. It should be noted that use of 1 mEq/L potassium dialysate was infrequent in our population, and it is therefore possible that we lacked sufficient power to detect an association between low potassium dialysate and arrhythmia rate.

We also identified a protective effect with the use of high concentration calcium dialysate which is fully consistent with registry-based studies suggesting an increase in sudden death risk with the use of low calcium dialysate [10]. The protective effect of low calcium dialysates we observed is harder to explain, but it could reflect use of lower calcium dialysates in individuals with significant hypercalcemia or hyperphosphatemia. Our finding that higher serum magnesium concentration is associated with a reduced rate of arrhythmia during and after dialysis is unique, but consistent with several recent registry analyses suggesting that the risk of all-cause, cardiovascular and sudden death is increased in hemodialysis patients with lower magnesium levels [21–23]. Given poor excretion of magnesium in end stage renal disease and the fixed nature of magnesium in standard dialysate baths, interest in the role of magnesium as a risk factor has been less than for other serum electrolytes. Our data suggests that further investigation of its role and consideration of studies manipulating the magnesium concentration in the dialysate or of oral supplementation should be considered. Unfortunately, dialysate magnesium concentration was not recorded in MiD and we could not directly assess this parameter.

Finally, we observed a decrease in arrhythmia rate with the use of dialysate sodium concentrations of 135–139 mEq/L (compared with 140 mEq/L) and bicarbonate concentrations > 35 mEq/L (compared with 35 mEq/L). The observed associations are intriguing given prior data implicating high dialysate sodium concentrations and acidosis as risk factors for mortality on dialysis [24–27].

Although our findings provide new insights into the pathogenesis of arrhythmia in hemodialysis patients, they differ from prior analyses identifying 1 mEq/L potassium and low calcium concentration dialysates as risk factors for sudden death [9, 10, 28]. Differences in the timing of the events captured (during dialysis to 8 h post vs. intradialytic only, or any time during follow-up), the number of patients exposed to low dialysate electrolyte concentrations, and the nature of the captured events (EKG-confirmed arrythmia vs. non-adjudicated sudden deaths that may have included deaths due to stroke, myocardial infarction, pulmonary embolism or vascular accidents) may account for the differential findings. Nevertheless, the differential findings suggest cautious interpretation and generalization of our data.

Several additional limitations should be kept in mind. First, we studied repeated prescription changes within a selected population of 66 patients that did not include any Hispanics rather than a larger more ethnically diverse population. Additionally, most individuals in MiD had been on dialysis longer than 1 year. The population studied is thus likely to over-represent survivors of the first year of dialysis during which the risk of sudden death is highest. Our findings should be generalized cautiously, particularly to incident patients. The sample size was small and extreme dialysate prescriptions (such as use of 1 mEq/L potassium dialysate) or electrolyte abnormalities were infrequent. These factors may have limited our power to detect associations with arrhythmia risk. In addition, we investigated multiple risk factors without correcting for multiple comparisons. Although correction for multiple comparisons, would have lowered the risk of type 2 error, it would have increased the risk of missing potentially important associations meriting further exploration. In light of the unique nature of our data and exploratory nature of our analysis, we chose not to utilize a correction procedure, and our findings should be considered hypothesis forming rather than definitive.

Our primary analysis examined all reviewer confirmed arrythmias. Power for the individual, post-hoc analyses of brady and tachyarrhythmias was more limited. However, results, were generally consistent with the primary analysis although larger intradialytic weight changes tended to decrease the likelihood of bradycardia as would be expected with volume depletion. Dedicated studies to better define the risk factors for individual arrythmia types are warranted. Finally, it should be noted that although the period starting with dialysis through eight hours post-dialysis was rationally selected as the most likely period to provide an informative analysis, this interval was selected following investigation of the periodicity of the underlying arrythmia rates. Whether alternative intervals would better represent the period in which heart rhythm is most likely to be directly and immediately influenced by the dialysis procedure.

In summary, we evaluated the occurrence of arrhythmia in hemodialysis patients during 6 months of intensive data capture and continuous electrocardiographic monitoring using implantable loop recorders with reviewer confirmation of identified arrhythmias. Arrhythmias were common, occurred in a temporal pattern related to thrice-weekly dialysis intervals, and were associated with potentially modifiable parameters such as serum magnesium, dialysate calcium and fluid and potassium flux during dialysis. Large studies to further develop these insights are needed, but our findings confirm the promise of individualizing the dialysis prescription as potentially important method of minimizing cardiac arrhythmias and sudden death in dialysis patients.

## Conclusions

In conclusion, peri-dialytic arrythmias are common in hemodialysis patients. They peak during the final 12 h of the inter-dialytic interval through the first 8 h after dialysis and may be more likely with use of low magnesium dialysate or jointly high rates of intra-dialytic potassium and fluid removal. Strengths of our analysis include the detailed capture of data and the continuous collection of heart rate data over 6 months. Limitations include the small sample size and infrequent occurrence of extreme electrolyte abnormalities or dialysate concentrations. Additional studies to validate and generalize our findings are warranted.

## Additional file

Additional file 1: **Table S1.** Baseline characteristics in individuals with and without reviewer confirmed arrhythmia during follow-up. **Table S2.** Laboratory values and dialysis prescription according to presence of reviewer confirmed arrhythmia. **Table S3.** Characteristics of sessions with and without reviewer confirmed bradycardia or asystole during follow-up. **Table S4.** Characteristics of sessions with and without reviewer confirmed tachycardias during follow-up. **Table S5.** Time-averaged laboratories according to the number of RCA observed during follow-up. **Table S6.** Time-averaged dialysis parameters according to the number of RCA during follow-up. (DOCX 43 kb)

## Abbreviations

CI: Confidence interval
CSA: Clinically significant arrythmia
ILR: Implantable loop recorder
IRR: Incidence rate ratio
mEq/L: Milliequivalent per liter
MiD: Monitoring in dialysis
RCA: Reviewer confirmed arrhythmias
SD: Standard deviation
