# Supplementary material for: Relationship between dialytic parameters and reviewer confirmed arrhythmias in hemodialysis patients in the monitoring in dialysis study
Source: BMC Nephrol. 2019 Mar 5;20:80. doi: 10.1186/s12882-019-1212-6 (PMC6402171; doi:10.1186/s12882-019-1212-6)
Supplement: Supplementary file 1 — Table S1. Baseline characteristics in individuals with and without reviewer confirmed arrhythmia during follow-up. Table S2. Laboratory values and dialysis prescription according to presence of reviewer confirmed arrhythmia. Table S3. Characteristics of sessions with and without reviewer confirmed bradycardia or asystole during follow-up. Table S4. Characteristics of sessions with and without reviewer confirmed tachycardias during follow-up. Table S5. Time-averaged laboratories according to the number of RCA observed during follow-up. Table S6. Time-averaged dialysis parameters according to the number of RCA during follow-up. (DOCX 43 kb) [file 12882_2019_1212_MOESM1_ESM.docx]

**Supplementary Table 1**. Baseline characteristics in individuals with and without reviewer confirmed arrhythmia during follow-up

| Characteristics | All Subjects  (N=66) | No RCA  (N=2) | RCA  (N=64) | P  Value |
| --- | --- | --- | --- | --- |
| Age (years) | 56.3 ± 12.2 (N=66) | 62.1 ± 18.3 (N=2) | 56.2 ± 12.1 (N=64) | 0.50 |
| Female | 30.3% (20/66) | 0.0% (0/2) | 31.3% (20/64) | >0.99 |
| Race |  |  |  | 0.35 |
| Asian | 34.8% (23/66) | 100.0% (2/2) | 32.8% (21/64) |  |
| Black | 53.0% (35/66) | 0.0% (0/2) | 54.7% (35/64) |  |
| Other | 1.5% (1/66) | 0.0% (0/2) | 1.6% (1/64) |  |
| White | 10.6% (7/66) | 0.0% (0/2) | 10.9% (7/64) |  |
| Systolic blood pressure | 140.8 ± 23.4 (N=66) | 125.0 ± 21.2  (N=2) | 141.3 ± 23.4  (N=64) | 0.34 |
| Diastolic blood pressure | 80 (70.0, 84.0) (N=66) | 65.0 (60.0, 70.0)  (N=2) | 80.0 (70.0, 84.0)  (N=64) | 0.16 |
| Weight (kg) | 81.7 (68.2, 95.2) (N=66) | 88.7 (74.8, 102.6)  (N=2) | 81.7 (67.9, 94.9)  (N=64) | 0.67 |
| BMI ≥ 40 | 9.1% (6/66) | 0.0% (0/2) | 9.4% (6/64) | >0.99 |
| Cause of ESRD |  |  |  |  |
| Diabetes | 42.4% (28/66) | 100.0% (2/2) | 40.6% (26/64) | 0.67 |
| Glomerulonephritis | 9.1% (6/66) | 0.0% (0/2) | 9.4% (6/64) |  |
| Hypertension | 37.9% (25/66) | 0.0% (0/2) | 39.1% (25/64) |  |
| Other | 10.6% (7/66) | 0.0% (0/2) | 10.9% (7/64) |  |
| ESRD Vintage (years) | 2.4 (1.2, 5.3)  (N=65) | 3.4 (2.2, 4.7)  (N=2) | 2.4 (1.1, 5.5)  (N=63) | 0.78 |
| Prior kidney transplant | 13.6% (9/66) | 0.0% (0/2) | 14.1% (9/64) | >0.99 |
| Previous peritoneal dialysis | 10.6% (7/66) | 0.0% (0/2) | 10.9% (7/64) | >0.99 |
| Vascular Access |  |  |  |  |
| AV Fistula | 69.2% (45/65) | 100.0% (2/2) | 68.3% (43/63) | >0.99 |
| AV Graft | 26.2% (17/65) | 0.0% (0/2) | 27.0% (17/63) |  |
| Catheter | 4.6% (3/65) | 0.0% (0/2) | 4.8% (3/63) |  |
| Diabetes | 63.6 %(42/66) | 100.0 (2/2) | 62.5% (40/64) | 0.53 |
| Hyperlipidemia | 60.6% (40/66) | 0.0% (0/2) | 62.5% (40/64) | 0.15 |
| Hypertension | 84.8% (56/66) | 100.0% (2/2) | 84.4% (54/64) | >0.99 |
| Ischemic heart disease | 48.5% (32/66) | 50.0% (1/2) | 48.4% (31/64) | >0.99 |
| History of MI | 9.1% (6/66) | 0.0% (0/2) | 9.4% (6/64) | >0.99 |
| Congestive heart failure | 25.8% (17/66) | 0.0% (0/2) | 26.6% (17/64) | >0.99 |
| Coronary artery bypass surgery | 13.6% (9/66) | 50.0% (1/2) | 12.5% (8/64) | 0.26 |
| Arrhythmia | 31.8% (21/66) | 0.0% (0/2) | 32.8% (21/64) | >0.99 |
| Smoking |  |  |  | >0.99 |
| Current | 7.6% (5/66) | 0.0% (0/2) | 7.8% (5/64) |  |
| Never | 69.7% (46/66) | 100.0% (2/2) | 68.8% (44/64) |  |
| Past | 22.7% (15/66) | 0.0% (0/2) | 23.4% (15/64) |  |
| LVEF | 55.0 (55.0, 60.0)  (N=65) | 55.0 (50.0, 60.0)  (N=2) | 55.0 (55.0, 60.0)  (N=63) | 0.65 |

Supplementary Table 1-Basline characteristics in individuals with and without reviewer confirmed arrhythmia (RCA) during follow-up. BMI-body mass index. ESRD-end stage renal disease. AV-arterio-venous. LVEF-Left ventricular ejection fraction. Data are mean ± SD, median (IQR), or % (n/N).

**Supplementary Table 2**. Laboratory values and dialysis prescription according to presence of reviewer confirmed arrhythmia

| **Characteristics** | **All Subjects**  (N=66) | **RCA**  (N=2) | **RCA**  **(**N=64) | **P**  **Value** |
| --- | --- | --- | --- | --- |
| Laboratory Values |  |  |  |  |
| Blood Urea Nitrogen (mg/dL)^*^ | 59.7 ± 17.8 | 54.0 ± 33.9 | 59.9 ± 17.5 | 0.65 |
| Sodium (mEq/L^*^ | 138.0 (135.0, 140.0) | 136.5 (133.0, 140.0) | 138.0 (135.0, 140.0) | 0.85 |
| Potassium (mEq/L)^*^ | 4.7 (4.2, 5.4) | 6.5 (4.2, 8.7) | 4.7 (4.3, 5.4) | 0.64 |
| CO2 (mEq/L)^*^ | 22.2 ± 3.7 | 17.5 ± 3.5 | 22.3 ± 3.6 | 0.07 |
| Calcium (mg/dL)^*^ | 8.7 ± 0.8 | 7.6 ± 1.1 | 8.7 ± 0.8 | 0.06 |
| Magnesium (mg/dL)^*^ | 2.3 (2.0, 2.7) | 2.9 (2.2, 3.7) | 2.3 (2.0, 2.6) | 0.43 |
| Phosphorous (mg/dL)^*^ | 5.1 (4.3, 6.3) | 5.7 (5.5, 5.8) | 5.1 (4.3, 6.3) | 0.48 |
| Hemoglobin (g/dL)^**^ | 10.7 (9.9, 11.4) | 10.5 (10.4, 10.6) | 10.8 (9.8, 11.4) | 0.89 |
| Albumin (g/dL)^*^ | 4.0 (3.8, 4.2) | 3.9 (3.7, 4.1) | 4.0 (3.8, 4.2) | 0.77 |
| spKt/V^*^ | 1.5 (1.2, 1.7) | 1.0 (0.9, 1.2) | 1.5 (1.2, 1.7) | 0.06 |
| Dialysis Parameters |  |  |  |  |
| Duration of hemodialysis (hrs) | 4.0 (3.5, 4.0) | 3.5 (3.0, 4.0) | 4.0 (3.5, 4.0) | 0.41 |
| Dry weight target (kg) | 80.5 (65.0, 94.0) | 85.5 (72.5, 98.5) | 80.5 (65.0, 93.0) | 0.68 |
| Kg Over dry weight target | 4.2 (2.7, 5.2) | 5.1 (4.1, 6.1) | 4.1 (2.6, 5.2) | 0.42 |
| Ultrafiltration rate (ml/kg/hr) | 10.9 (7.4, 15.9) | 15.6 (13.9, 17.2) | 10.9 (7.3, 15.9) | 0.22 |
| Sodium modeling | 13.6% (9/66) | 50.0% (1/2) | 12.5% (8/64) | 0.26 |
| High flux dialyzer | 63.6% (42/66) | 0.0% (0/2) | 65.6% (42/64) | 0.13 |
| Membrane reuse | 27.3% (18/66) | 50.0% (1/2) | 26.6% (17/64) | 0.47 |
| Cellulose membrane | 7.6% (5/66) | 0.0% (0/2) | 7.8% (5/64) | >0.99 |
| Dialysate temperature (℃) | 37.0 (37.0, 37.0) | 36.8 (36.5, 37.0) | 37.0 (37.0, 37.0) | 0.18 |
| Dialysate sodium (mEq/L)^†^ | 140.0 (140.0, 140.0) | 140.0 (140.0, 140.0) | 140.0 (140.0, 140.0) | 0.50 |
| Dialysate potassium (mEq/L) | 2.0 (2.0, 2.0) | 2.0 (2.0, 2.0) | 2.0 (2.0, 2.0) | 0.57 |
| Dialysate bicarbonate (mEq/L) | 35.0 (33.0, 36.0) | 35.0 (35.0, 35.0) | 35.0 (32.5, 36.5) | >0.99 |
| Dialysate calcium (mEq/L) | 2.5 (2.5, 2.5) | 1.6 (1.6, 1.6) | 2.5 (2.5, 2.5) | 0.04 |

Table 2-Distribution of baseline lab tests and dialysis parameters. CO2-total carbon dioxide (bicarbonate). SpKt/V-single pool Kt/V according to Daugirdas formula. CRP-HS-high sensitivity CRP. PTH-parathyroid hormone. Hrs-hours. mEq-milliequivalent. mg-milligram. dL-deciliter. L-liter. pg-picogram. Celsius. mL-milliliter. Kg-kilogram. CRP, PTH and BNP were measured in US patients only^. *^Available in 59 patients, 2 without RCA and 57 with RCA. ^**^Available in 56 patients, 2 without RCA and 54 with RCA. ^‡^ Available in 42 patients, 11 without CSA and 31 with CSA †Dialysate sodium was available in 61 patients overall, 2 without RCA and 59 with RCA.

**Supplementary Table 3.** Characteristics of sessions with and without reviewer confirmed bradycardia or asystole during follow-up

| **Characteristic** | **Session without Brady** | **Session with Brady** | **P Value** |
| --- | --- | --- | --- |
| Duration of hemodialysis (hrs) | 4711 3.9 ± 0.5 | 47 3.5 ± 0.4 | 0.20 |
| Dry weight (kg) | 4707 85.4 ± 28.4 | 47 85.4 ± 15.9 | 0.19 |
| Percent over dry weight (%) | 4694 3.9 ± 2.4 | 47 3.4 ± 3.2 | 0.76 |
| Kilogram over dry weight | 4694 3.2 ± 1.9 | 47 2.7 ± 2.3 | 0.84 |
| Ultrafiltration rate (ml/kg/hr) | 4711 9.9 ± 4.8 | 47 8.3 ± 3.9 | 0.87 |
| Intradialytic decrease in weight (kg) | 4694 2.7 ± 1.3 | 47 2.0 ± 1.0 | 0.05 |
| Pre-dialysis potassium (mEq/L) | 1647 4.9 ± 0.8 | 20 5.1 ± 0.6 | 0.47 |
| Intradialytic potassium change (mEq/L) | 1597 -1.2 ± 0.8 | 20 -1.4 ± 0.6 | 0.88 |
| Pre-dialysis calcium (mEq/L) | 1656 8.7 ± 0.9 | 20 8.5 ± 0.8 | 0.91 |
| Intra-dialytic calcium change (mEq/L) | 1600 0.7 ± 1.0 | 20 0.6 ± 0.9 | 0.51 |
| Pre-dialysis magnesium (mg/dL) | 1656 2.4 ± 0.5 | 20 2.2 ± 0.4 | 0.15 |
| Intradialytic magnesium change (mg/dL) | 1605 -0.3 ± 0.3 | 20 -0.3 ± 0.3 | 0.99 |
| Pre-dialysis phosphorus (mg/dL) | 1654 5.3 ± 1.7 | 20 5.0 ± 1.6\ | 0.33 |
| Intradialytic phosphorus change (mg/dL) | 1597 -3.0 ± 1.5 -3.0 (-4.0, -2.0) | 20 -3.0 ± 1.3 -2.8 (-3.4, -2.3) | 0.30 |
| Pre-dialysis bicarbonate (mEq/L) | 1655 22.2 ± 4.0 | 20 22.0 ± 4.3\ | 0.09 |
| Intradialytic bicarbonate change (mEq/L) | 1603 4.9 ± 3.3 | 20 5.7 ± 3.8 | 0.27 |
| Pre-dialysis sodium (mEq/L) | 1656 136.8 ± 4.3 | 20 137.5 ± 3.5 | 0.20 |
| Intradialytic sodium change (mEq/L) | 1600 0.5 ± 4.0 | 20 1.4 ± 3.1 | 0.33 |
| Nadir intradialytic systolic blood pressure (mm Hg) | 4705 120.0 (104.0, 136.0) | 47 115.0 (103.0, 129.0) | 0.04 |
| Nadir intradialytic diastolic blood pressure (mm Hg) | 4705 66.0 (56.0, 74.0) | 47 59.0 (51.0, 63.0) | 0.10 |
| **Dialysis Prescription Parameters** |  |  |  |
| Dialysate temperature |  |  | --* |
| 36-36.9℃ | 12.7% (585/4612) | 4.3% (2/47) |  |
| ≥37℃ | 87.3% (4027/4612) | 95.7% (45/47) |  |
| Dialysate potassium |  |  | 0.81 |
| 2.0 mEq/L | 81.5% (3712/4555) | 76.6% (36/47) |  |
| 3.0 mEq/L | 18.5% (843/4555) | 23.4% (11/47) |  |
| Dialysate calcium |  |  | 0.07 |
| <2.0 mEq/L | 20.2% (951/4702) | 10.6% (5/47) |  |
| 2.0-2.4 mEq/L | 1.7% (82/4702) | 0.0% (0) |  |
| 2.5 mEq/L | 55.6% (2612/4702) | 68.1% (32/47) |  |
| >2.5 mEq/L | 22.5% (1057/4702) | 21.3% (10/47) |  |
| Dialysate sodium |  |  | 0.25 |
| ≤135 mEq/L | 13.2% (568/4314) | 6.4% (3/47) |  |
| 136-139 mEq/L | 9.9% (427/4314) | 4.3% (2/47) |  |
| 140 mEq/L | 76.9% (3319/4314) | 89.4% (42/47) |  |
| Sodium modeling | 13.4% (628/4695) | 2.1% (1/47) | 0.10 |
| Dialysate bicarbonate |  |  | 0.05 |
| ≤28 mEq/L | 5.3% (243/4595) | 2.1% (1/47) |  |
| 29-34 mEq/L | 23.4% (1076/4595) | 21.3% (10/47) |  |
| 35 mEq/L | 38.3% (1761/4595) | 8.5% (4/47) |  |
| >35 mEq/L | 33.0% (1515/4595) | 68.1% (32/47) |  |

Data are presented as number of sessions for which data is availalbe over mean ± standard deviation or as % (n/N)>. Hrs-hours, Kg-kilogram, mL=milliliter, mg/dL-milligram per deciliter. mm Hg-millimeters of mercury. * non-estimable due to non-converging model parameters.

**Supplementary Table 4.** Characteristics of sessions with and without reviewer confirmed tachycardias during follow-up

| **Characteristic** | **Session without Tachycardia** | **Session with Tachycardia** | **P Value** |  |
| --- | --- | --- | --- | --- |
| Duration of hemodialysis (hrs) | 4169 3.8 ± 0.5 | 590 3.9 ± 0.6 | 0.84 |  |
| Dry weight (kg) | 4165 84.8 ± 27.9 | 590 90.1 ± 30.3 | 0.8239 |  |
| Percent over dry weight (%) | 4153 3.9 ± 2.4 | 589 4.2 ± 2.4 | 0.05 |  |
| Kilogram over dry weight | 4153 3.1 ± 1.9 | 589 3.6 ± 2.1 | 0.07 |  |
| Ultrafiltration rate (ml/kg/hr) | 4169 9.9 ± | 590 10.2 ± 4.6 | 0.01 |  |
| Intradialytic decrease in weight (kg) | 4153 2.6 ± 1.3 | 589 3.0 ± 1.3 | 0.002 |  |
| Pre-dialysis potassium (mEq/L) | 1419 4.9 ± 0.8 | 248 5.0 ± 0.8 | 0.80 |  |
| Intradialytic potassium change (mEq/L) | 1375 -1.2 ± 0.8 | 242 -1.3 ± 0.8 | 0.11 |  |
| Pre-dialysis calcium (mEq/L) | 1427 8.7 ± 0.9 | 249 8.8 ± 0.9 | 0.41 |  |
| Intra-dialytic calcium change (mEq/L) | 1377 0.7 ± 1.1 | 243 0.6 ± 0.9 | 0.53 |  |
| Pre-dialysis magnesium (mg/dL) | 1427 2.4 ± 0.5 | 249 2.2 ± 0.4 | 0.66 |  |
| Intradialytic magnesium change (mg/dL) | 1381 -0.3 ± 0.3 | 244 -0.3 ± 0.3 | 0.85 |  |
| Pre-dialysis phosphorus (mg/dL) | 1425 5.3 ± 1.7 | 249 5.1 ± 1.7 | 0.2354 |  |
| Intradialytic phosphorus change (mg/dL) | 1373 -3.0 ± 1.5 -3.0 (-4.0, -2.0) | 244 -3.0 ± 1.5 -2.9 (-3.9, -2.0) | 0.22 |  |
| Pre-dialysis bicarbonate (mEq/L) | 1426 22.1 ± 3.9 | 249 22.7 ± 4.1 | 0.69 |  |
| Intradialytic bicarbonate change (mEq/L) | 1379 4.9 ± 3.3 | 244 4.6 ± 3.5 | 0.83 |  |
| Pre-dialysis sodium (mEq/L) | 1427 136.6 ± 4.3 | 249 137.9 ± 4.1 | 0.14 |  |
| Intradialytic sodium change (mEq/L) | 1377 0.5 ± 4.1 | 243 0.1 ± 3.4 | 0.45 |  |
| Nadir intradialytic systolic blood pressure (mm Hg) | 4163 120.0 (105.0, 138.0) | 590 113.0 (99.0, 130.0) | 0.29 |  |
| Nadir intradialytic diastolic blood pressure (mm Hg) | 4163 66.0 (56.0, 74.0) | 590 62.0 (54.0, 72.0) | 0.04 |  |
| **Dialysis Prescription Parameters** |  |  |  |  |
| Dialysate temperature |  |  | <0.0001 |  |
| 36-36.9℃ | 13.3% (542/4079) | 7.7% (45/581) |  |  |
| ≥37℃ | 86.7% (3537/4079) | 92.3% (536/581) |  |  |
| Dialysate potassium |  |  | 0.06 |  |
| 2.0 mEq/L | 82.9% (3335/4023) | 71.4% (414/580) |  |  |
| 3.0 mEq/L | 17.1% (688/4023) | 28.6% (166/580) |  |  |
| Dialysate calcium |  |  | <0.0001 |  |
| <2.0 mEq/L | 22.4% (930/4161) | 4.4% (26/589) |  |  |
| 2.0-2.4 mEq/L | 1.9% (81/4161) | 0.2% (1/589) |  |  |
| 2.5 mEq/L | 53.3% (2219/4161) | 72.2% (425/589) |  |  |
| >2.5 mEq/L | 22.4% (931/4161) | 23.3% (137/589) |  |  |
| Dialysate sodium |  |  | >0.99 |  |
| ≤135 mEq/L | 13.2% (504/3818) | 12.3% (67/543) |  |  |
| 136-139 mEq/L | 10.7% (410/3818) | 3.5% (19/543) |  |  |
| 140 mEq/L | 76.1% (2904/3818) | 84.2% (457/543) |  |  |
| Sodium modeling | 14.0% (582/4155) | 8.0% (47/588) | 0.40 |  |
| Dialysate bicarbonate |  |  | 0.20 |  |
| ≤28 mEq/L | 5.3% (216/4057) | 4.8% (28/586) |  |  |
| 29-34 mEq/L | 23.9% (968/4057) | 20.3% (119/586) |  |  |
| 35 mEq/L | 38.3% (1552/4057) | 36.3% (213/586) |  |  |
| >35 mEq/L | 32.6% (1321/4057) | 38.6% (226/586) |  |  |

Data are presented as number of sessions over mean ± standard deviation or as % (n/N)>. Hrs-hours, Kg-kilogram, mL=milliliter, mg/dL-milligram per deciliter. mm Hg-millimeters of mercury.

**Supplementary Table 5**. Time-averaged laboratories according to the number of RCA observed during follow-up

| **Characteristics** | **All Subjects**  (N=66) | **Number of RCA During Follow-up** | | | | **P Value** |
| --- | --- | --- | --- | --- | --- | --- |
|  |  | **≤10**  (N=16) | **11-49**  (N=16) | **50-239**  (N=17) | **≥240**  (N=17) |  |
| Blood urea nitrogen (mg/dL) | 56.2 ± 12.9 | 56.8 ± 12.5 | 48.9 ± 10.7 | 60.6 ± 11.5 | 58.0 ± 14.4 | 0.05 |
| Creatinine (mg/dL) | 9.8 ± 3.1 | 9.1 ± 2.9 | 9.5 ± 3.6 | 10.3 ± 3.0 | 10.2 ± 3.2 | 0.66 |
| Sodium (mEq/L) | 137.0 (134.0, 139.0) | 136.3 (133.0, 137.0) | 137.5 (135.0, 139.3) | 138.0 (137.0, 139.0) | 138.0 (136.0, 140.0) | 0.14 |
| Potassium (mEq/L) | 4.8 (4.4, 5.1) | 5.1 (4.6, 5.3) | 4.6 (4.2, 4.8) | 4.7 (4.3, 4.9) | 5.0 (4.8, 5.3) | 0.04 |
| CO2 (mEq/L) | 22.3 ± 3.3 | 21.3 ± 3.2 | 22.5 ± 2.5 | 22.5 ± 3.5 | 22.7 ± 3.8 | 0.63 |
| Calcium (mg/dL) | 8.7 ± 0.7 | 8.6 ± 0.7 | 8.7 ± 0.7 | 8.6 ± 0.6 | 8.9 ± 0.8 | 0.54 |
| Magnesium (mg/Dl) | 2.3 (2.0, 2.6) | 2.6 (2.3, 2.8) | 2.3 (2.0, 2.6) | 2.3 (2.2, 2.6) | 2.2 (2.0, 2.4) | 0.01 |
| Phosphorous (mg/dL) | 5.1 (4.4, 6.1) | 4.9 (4.5, 6.1) | 4.7 (3.6, 5.2) | 5.7 (4.8, 6.7) | 5.2 (4.2, 6.6) | 0.14 |
| Hemoglobin (g/dL) | 10.7 (10.1, 11.4) | 10.3 (9.9, 10.8) | 10.6 (9.7, 11.5) | 10.3 (10.1, 11.1) | 11.0 (10.7, 11.4) | 0.16 |

Data are presented as mean ± SD or median (25^th^, 75^th^ percentile); Mg/dL-milligram/deciliter. mEq/L-mEq/L, g/dL-gram/deciliter.

**Supplementary Table 6**. Time-averaged dialysis parameters according to the number of RCA during follow-up

| **Characteristics** | **All Subjects**  (N=66) | **Number of RCA During Follow-up** | | | | **P Value** |
| --- | --- | --- | --- | --- | --- | --- |
|  |  | **≤10**  (N=16) | **11-49**  (N=16) | **50-239**  (N=17) | **≥240**  (N=17) |  |
| Dry weight target (kg) | 80.0 (66.0, 94.0) | 76.0 (66.3, 88.3) | 81.3 (61.5, 86.8) | 78.0 (65.5, 116.0) | 83.5 (76.5, 107.5) | 0.42 |
| Kg over dry weight target (kg) | 3.0 (2.4, 3.7) | 3.0 (2.0, 3.5) | 2.6 (1.8, 3.1) | 3.2 (2.6, 3.6) | 3.5 (2.6, 4.5) | 0.11 |
| Ultrafiltration rate (ml/kg/hr) | 9.0 (7.4, 11.9) | 8.9 (7.2, 11.6) | 10.0 (6.0, 11.0) | 8.6 (8.0, 10.9) | 9.3 (7.5, 13.6) | 0.72 |
| Dialysate temperature (℃) | 37.0 (37.0, 37.0) | 37.0 (36.8, 37.0) | 37.0 (37.0, 37.0) | 37.0 (37.0, 37.0) | 37.0 (37.0, 37.0) | 0.52 |
| Dialysate potassium (mEq/L) | 2.0 (2.0, 2.0) | 2.0 (2.0, 2.0) | 2.0 (2.0, 2.0) | 2.0 (2.0, 2.0) | 2.0 (2.0, 3.0) | 0.05 |
| Dialysate calcium (mEq/L) | 2.5 (2.5, 2.5) | 2.5 (1.6, 2.8) | 2.5 (2.1, 3.0) | 2.5 (2.5, 2.5) | 2.5 (2.5, 2.5) | 0.66 |
| Dialysate sodium (mEq/L)* | 140.0 (140.0, 140.0) | 140.0 (138.0, 140.0) | 140.0 (140.0, 140.0) | 140.0 (140.0, 140.0) | 140.0 (140.0, 140.0) | 0.21 |
| Dialysate bicarbonate (mEq/L) | 35.0 (33.0, 37.0) | 35.0 (32.5, 35.0) | 35.0 (32.0, 37.0) | 35.0 (35.0, 37.0) | 35.0 (35.0, 40.0) | 0.41 |

Hrs-hours. Kg-kilogram, ml-milliliter, hr-hour, mEq/L-milli-equivalent/Liter. * Dialysate sodium was available in only 14/16 individuals with <10 RCA, and 14/16 individuals with 10-49 RCA.
